# Evaluation of Virucidal Efficacy of Human Norovirus Using Combined Sprayed Slightly Acidic Electrolyzed Water and Ultraviolet C-Light-Emitting Diode Irradiation Treatment Based on Optimized Capture Assay for Quantitative RT-qPCR

**DOI:** 10.3389/fmicb.2022.841108

**Published:** 2022-04-25

**Authors:** Hyeyeon Song, Yun-Mi Dang, Sanghyun Ha, Ji-Hyoung Ha

**Affiliations:** Hygienic Safety and Distribution Research Group, World Institute of Kimchi, Gwangju, South Korea

**Keywords:** human norovirus, response surface methodology, slightly acidic electrolyzed water, spray disinfection, ultraviolet light-emitting diode

## Abstract

Slightly acidic electrolyzed water (SAEW), an effective non-thermal virucidal treatment, is used widely to prevent infectious viral cross-contamination. Surface disinfection technologies using ultraviolet C-light-emitting diode (UVC-LED) irradiation have recently attracted considerable attention. The SAEW sprayer technique is an efficient approach to preventing the spread of infectious viral pathogens in the public healthcare sector. Therefore, we investigated a small-scale system comprising sprayed SAEW disinfection combined with UVC-LED irradiation to inactivate the human norovirus (HuNoV) in the environment. A stainless-steel surface was inoculated with a HuNoV genogroup II genotype 4 (GII.4) to achieve maximum reduction values of 3.21 log10 genomic copies. For optimal disinfection conditions, the response surface methodology based on the Box–Behnken design revealed that the specific treatment conditions for inactivation of HuNoV GII.4 were an SAEW droplet volume of 180 μL, 30 ppm available chlorine concentration of SAEW, and a UVC-LED exposure dose of 2 mJ/cm^2^. The results indicate that the combined disinfection treatment could efficiently prevent the spread of HuNoVs in environment. Furthermore, the quadratic polynomial equations of the 3-D response surface can be employed to predict the effects of combined disinfection treatment on HuNoV contamination on environmental surfaces. Therefore, sprayed SAEW disinfection combined with UVC-LED irradiation proposed in this study may offer insights for designing optimal control strategies and techniques to prevent the transmission of infectious diseases, particularly HuNoV.

## Introduction

Human norovirus (HuNoV), which has characteristics such as high viral load excretion, tenacious environmental sustainability, rapid propagation, and short-term immunity, causes sporadic cases of viral gastroenteritis. Owing to its high contagiousness and rapid spread around the world, it infects humans regardless of gender or age (Hartmann et al., 2015). HuNoV infection is the most common cause of acute gastroenteritis, causing approximately 680 million cases per year (CDC, 2021). Particularly, the Centers for Disease Control and Prevention report that one in five cases of acute gastroenteritis (inflammation of the intestines or stomach), which causes vomiting and diarrhea, is caused by HuNoV infections worldwide. Furthermore, in most developing countries, approximately 200 million outbreaks are recorded among children under the age of 5 years, and approximately 50,000 deaths are estimated every year.

The virus causing gastroenteritis is known to spread mainly via environmental or waterborne routes, direct transmission between people, or fecal–oral routes (Dewey-Mattia et al., 2018). Among the various transmission routes, the airborne fomite route includes aerosol droplets. Several studies demonstrate that viruses in the form of aerosol particles are a remarkable characteristic of HuNoV transmission (de Graaf et al., 2016). Bonifait et al. (2015) demonstrates that intact HuNoV particles can be aerosolized from vomit and accumulate in the upper respiratory tract during respiration and in saliva of patients. By quantifying HuNoV RNA in air samples, it was demonstrated that the HuNoV epidemic was caused by aerosol contamination of the virus in healthcare settings (Bonifait et al., 2015). In addition, Simmons et al. (2020) demonstrate that HuNoV contamination on various surfaces in airplane cabins is caused by aerosol particles via RNA quantification. Bonifait et al. (2015) emphasize that NoVs remain contagious after aerosolization in environmental fomite, where the concentrations of airborne viral particles are ample to affect humans. Aerosol droplets containing NoV particles can accumulate on certain fomites, thereby directly causing contact transmission. In such cases, viral infection occurs by touching the nose and/or mouth with hands contaminated by viral particles after touching fomites. In numerous previous studies, secondary contamination of environmental fomites by HuNoVs aerosol is proven to be a significant route of infection (Bonifait et al., 2015; Kirby et al., 2016; Simmons et al., 2020).

Recently, owing to its considerable antimicrobial effects on pathogenic microorganisms and the non-formation of disinfection by-products, slightly acidic electrolyzed water (SAEW) treatment has been considered a successful method of disinfection in healthcare settings. Particularly, among the common chemical disinfectant materials, including ethanol, chlorine and its compounds, iodine, electrolyzed water, organic compounds, hydrogen peroxide, and quaternary ammonium compounds, SAEW shows broad-spectrum virucidal efficacy against a range of viral pathogens, such as non-enveloped viruses. Several studies also suggest that SAEW disinfection has virucidal activity against pathogenic viruses, including hepatitis C and hepatitis B viruses (Morita et al., 2000; Tagawa et al., 2000; Sakurai et al., 2003). SAEW virucidal efficacy against cultivable HuNoV surrogate (e.g., murine norovirus 1; MNV-1) is verified, and sufficient virucidal efficacy against HuNoV genogroup II genotype 4 (GII.4) Sydney on stainless-steel surfaces and in suspension is reported in several studies (Montazeri et al., 2017; Moorman et al., 2017). Based on previous scientific studies, the Japanese Ministry of Health, Labor, and Welfare (June 2002) approved SAEW as a food additive to eliminate pathogenic microbial populations in foods. Furthermore, the U.S. Environmental Protection Agency authorized the use of electrolyzed water generators for inactivation of pathogens in the environmental sectors.

The use of ultraviolet C-light-emitting diode (UVC-LED) has emerged as the optimal alternative approach and is recognized as a durable UV source to replace mercury UV lamps. It is environment-friendly and relatively cost-effective (Song et al., 2016) since conventional mercury UV lamps can cause chemical pollution, have a short bulb lifetime, and have low energy efficiency (Vilhunen and Sillanpää, 2010). In addition to preventing mercury disposal, UVC-LED irradiation has numerous advantages, including lesser warmup time, better device operation, lower power consumption, higher stability, and lower operating voltage than that of mercury UV lamps (Khan, 2006). Therefore, UVC-LED treatment (ranging between 100 and 280 nm) is broadly used in various sectors to block infection transmission and ensure public health. Across the entire UVC radiation spectrum, UVC-LEDs of wavelengths 269–276 nm are highly effective for inactivating infectious viruses in water and air and on any type of environmental surface (Bhardwaj J. et al., 2021).

To date, no studies have investigated the optimal disinfection characteristics of the hurdle approach combining the sprayed SAEW treatment and UVC-LED irradiation against HuNoV on stainless-steel surfaces. The objective of the present study was to determine the optimal conditions for inactivating HuNoV droplets on stainless-steel surfaces. To verify the optimal parameters and correlation between experimental variables, including the UV dose (mJ/cm^2^), available chlorine concentration (ACC) of SAEW (ppm), and sprayed SAEW amount (μL), a multivariate statistical analysis based on a combination of the response surface methodology (RSM) and the Box–Behnken design (BBD) technique was applied.

## Materials and Methods

### Viral Stock Preparation

The stock sample of HuNoV GII.4 was diluted in RNase-free water (Qiagen, Hilden, Germany) and vortexed briefly. The viral supernatant suspension (approximately 6.58 log_10_ genomic copies/μL) was stored in 0.5-mL aliquots at −70°C until use. HuNoV GII.4 was obtained from the Catholic University of Korea (Seoul, South Korea).

### Inoculation on Stainless-Steel Chips

For spiking the prepared viral suspension onto the stainless-steel surface, which was cut from an SUS ANSI 304SS 2B stainless-steel plate sheet (Posco Co., Ltd., Pohang, South Korea), all stainless-steel chips (3 × 3 cm pieces) were disinfected by soaking thoroughly with 20,000 ppm sodium hypochlorite and further wiping with 70% ethanol. Finally, all chip samples were sterilized in an ultrasonic cleaning solution (Fisher Scientific, Pittsburgh, PA, United States) for 5 min and rinsed in RNase-free water. Prior to the experiment, each sterilized chip was further wrapped with UVC disinfection-treated aluminum foil packed in a glass container to decontaminate in an autoclave at 121°C. To obtain a HuNoV GII.4 suspension (300 μL), 30 μL of HuNoV sample was added to 270 μL of RNase-free water and inoculated with the approximately 5.58 log_10_ genomic copies/μL viral suspension on the prepared surface. To dry the spiked HuNoV GII.4 suspension from the surface, all artificially contaminated chips were stored in a laminar flow hood for 1 h at 18 ± 3°C.

### Experimental Design and Data Analysis

This study used the BBD combined with RSM to analyze and investigate the optimal parameters for virucidal effects of HuNoV GII.4 based on different variables, including the amount of sprayed EW droplets, ACC of sprayed EW, and UVC-LED dose as suggested by Song et al. (2018). In this study, we selected a three-level BBD, three-parameter set of points located at the midpoints at each end, and the midpoints of the replicated centroids to obtain a quadratic polynomial regression model. The experimental design was established using Minitab 19 (Minitab LLC., version 19, Systat Software, State College, PA, United States). We also investigated the interrelationship between the experimental parameters for the combined disinfection treatment of HuNoV. A laboratory scale SAEW generator (Purester m-Clean; Morinaga Milk Industry Co., Ltd., Tokyo, Japan) was used for SAEW generation, at 4.2 A and 11.7 V, and a flow rate of 10.0 L/min. The ACC was measured based on the colorimetric method using a digital chlorine test kit (RC-3F; Kasahara Chemical Co., Saitema, Japan). Maximum SAEW had an ACC of 30.16 ± 0.34 ppm. After production, the SAEW was placed in a custom stainless-steel tank for disinfection. The independent variables were the amount of SAEW droplets (60, 120, and 180 μL), ACC of the sprayed SAEW (4, 17, and 30 ppm), and UVC-LED dose (1.08, 2.16, and 3.24 mJ/cm^2^). Statistical processing of data was conducted at three levels with the three independent parameters: −1 and + 1 indicate high and low levels, respectively, and 0 denotes the midpoint for determining the experimental error (Table 1). The following second order polynomial regression model was employed to analyze the influence of the three independent variables on the combined disinfection treatment of HuNoV inactivation:


(1) $$Y_{H\,u\,N\,o\,V}=\beta_{0}+\sum_{i=1}^{k}\beta_{i}\,{\text{X}}_{i}+\sum_{i=1}^{k}\beta_{i\,i}\,{\text{X}}_{i}^{2}+\sum_{i_{i=1}}^{k}\sum_{j>1}^{k}\beta_{i\,j}\,{\text{X}}_{i}\,{\text{X}}_{j}+\varepsilon ,$$

**TABLE 1:** Parameters and levels used in the Box–Behnken desig (BBD) matrix for the inactivation of the human norovirus (HuNoV).

Response code		Meaning							

*Y_*HuNoV*_*		HuNoV reduction value (Log_10_ genomic copies)							

		Factors							
			
Level		X_1_ SAEW amount			X_2_ ACC of SAEW			X_3_ UVC-LED dose	

Low (−1)		60			4			c	
Intermediate (0)		120			17			2	
High (+ 1)		180			30			3	
Run	Coded values			Actual values			Response values		
			
	X_1_	X_2_	X_3_	X_1_	X_2_	X_3_	Observed		Predicted
1	1	−1	−1	60	4	2	1.49		1.55
2	1	0	−1	120	4	3	1.64		1.58
3	1	0	0	120	17	2	2.03		2.01
4	1	−1	0	60	17	1	1.70		1.63
5	1	1	1	180	30	2	3.21		2.95
6	1	−1	1	60	30	2	2.45		2.46
7	1	0	−1	120	4	1	0.95		0.96
8	1	1	0	180	17	3	2.18		2.25
9	1	0	1	120	30	3	2.59		2.57
10	1	0	0	120	17	2	2.01		2.01
11	1	1	0	180	17	1	1.86		1.86
12	1	−1	0	60	17	3	2.07		2.07
13	1	1	−1	180	4	2	1.48		1.47
14	1	0	0	120	17	2	2.00		2.01
15	1	0	1	120	30	1	2.29		2.35

*Three-parameter BBD matrix with experimental and predicted response values for the HuNoV reduction.*

where *Y*_*HuNoV*_ is the HuNoV inactivation response; β_0_ is the constant term; and β_*i*_, β_*ii*_, and β_*ij*_ are the linear, quadratic, and interaction coefficients of the regression, respectively. Independent variables are denoted *X*_*i*_ and *X*_*j*_, and ε is the error. A statistical analysis of variance (ANOVA) based on the BBD was conducted using Minitab 19 (Minitab LLC.) to demonstrate the suitability and fitness of the regression model coefficient. Various statistical parameters, including the lack-of-fit test, multiple determination coefficient (*R*^2^) tests, and ANOVA were applied to analyze the second order regression model significance.

### Sequential Disinfection Using Sprayed Slightly Acidic Electrolyzed Water and Ultraviolet C-Light-Emitting Diode

**FIGURE 1:**
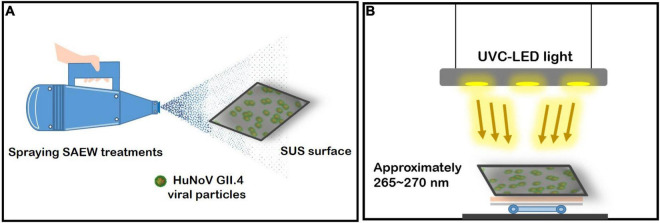
Schematic representation of the experimental devices. **(A)** Spraying slightly acidic electrolyzed water (SAEW) treatment and ultraviolet C light-emitting diode (UVC-LED) irradiation system for norovirus (HuNoV), and **(B)** UV irradiation performance under UVC-LED light.

The combined disinfection treatment using sprayed SAEW and UVC-LED is illustrated in Figure 1. SAEW (ACC, 30 ppm) with a pH range of 5.40 and an oxidation reduction potential of 1,230 mV was obtained by electrolysis of 6.3% hydrochloric acid in a chamber without a membrane using electrolyzed water equipment (Purester m-Clean; Morinaga Engineering Co., Ltd., Tokyo, Japan) set at 11.5 V and 4.5 A. The sprayed SAEW droplet (droplet size, approximately 70 ± 20 μm; maximum discharge capacity, 1,200 mL/60 s) was produced using an electric ultralow volume (ULV) sprayer (Atomer-2 RA04HS, JY Industry Co., Seoul, South Korea). A modified quantitative disk carrier test (ASTM E2197), a globally recognized disinfectant testing protocol, was employed in the present study. For SAEW droplet generation, virucidal efficacy experiments were conducted in a 2.5 m^3^ chamber, in which the air ventilation had been blocked to completely control airflow for the spraying test. The chips inoculated with HuNoV GII.4 allowed contact between HuNoV GII.4 and SAEW droplets following spraying-disinfection with SAEW droplets. Concurrently, UV-LED irradiation (wavelength of approximately 265–270 nm) experiments were performed to inactivate HuNoV GII.4. Artificially contaminated individual stainless-steel chips were treated in the following designed experimental set: The amount of SAEW droplets (60, 120, and 180 μL), ACC of the sprayed SAEW (4, 17, and 30 ppm), and UVC-LED dose (1.08, 2.16, and 3.24 mJ/cm^2^). The fronts of the disinfected chip samples sprayed with SAEW were irradiated by UVC-LEDs with intensities of 18 μW/cm^2^ for 60, 120, and 180 s. Triplicate disinfection experiments were conducted to evaluate the virucidal activity.

### Enumeration of Human Norovirus Populations

#### Recovery of HuNoVs

To concentrate, elute, and quantify HuNoV GII.4, individual treated chip samples were prepared as described in a previous experiment (Moon et al., 2020). Immediately following sprayed SEAW disinfection combined with UVC-LED irradiation, the Enviro-Max Environmental sampling cotton swab kit (Puritan Medical Products Company LLC, ME, United States) was used to collect the viral particles of HuNoV GII.4 from each stainless-steel chip. The virucidal activation was terminated immediately after the disinfection treatment was completed so that the SAEW remaining in the coupon did not damage the viral particles and affect the recovery rate of the infectious virus particles, and then the virus was captured. Each chip was swabbed horizontally, vertically, and diagonally on both sides of the cotton swab, 20 times in each direction. Subsequently, 20 mL of 0.05 M Glycine-0.14 M NaCl (pH 7) solution was employed to elute the viral particle of HuNoV GII.4 from the Enviro-Max Environmental sampling cotton swab, followed by immersion in a transport tube at 18°± 3°C for 3 min with constant shaking (approximately 70 rpm) and intense vortexing for 60 s. To obtain the whole liquid from the cotton swab, it was squeezed to completely release the liquid against the inside of the tube. The remaining HuNoV GII.4 on each stainless-steel chip was repeatedly wiped using a new 20-mL elution buffer with a silver swab, and the 20-mL secondary suspension was added to a 50-mL tube containing 20 mL primary elution buffer.

#### Optimized Quantitative RT-qPCR of HuNoVs

An anionic polymer-conjugated magnetic beads separation (MBS) assay with Viro-adembeads (Ademtech, Pessac, France), which were prepared by conjugation of poly(methyl vinyl ether-maleic anhydride) [poly(MVE-MA)]) was employed to recover and capture the HuNoV GII.4 from the obtained test suspension to concentrate the HuNoV viral particles. In the MBS technique, to concentrate the viral particles of HuNoV GII.4, 30 mL of the viral particle suspension was mixed with 50 μL of anionic polymer-coated MBS (final concentration: 15 mg/mL) in a 50-mL conical tube and agitated for 60 min at 18°± 3°C. The LifeSep MBS stand (Sigma-Aldrich) was employed to distinguish the magnetic beads and the supernatant in the captured HuNoV viral particle suspension. The magnetic beads captured HuNoV viral particles and were resuspended in 280 μL of RNase-free water. Immediately following inactivation tests, RNA isolation and virucidal analysis were performed to prevent the influence of sample freezing. Optimal quantification analysis of viral particles by treatment with SAEW and UVC-LED against HuNoV GII.4 was conducted as demonstrated in a previous study: an MBS/real time-reverse transcriptase polymerase chain reaction (RT-qPCR) method with a pretreatment combining propidium monoazide (PMA) (MBS/PMA/RT-qPCR) (Moon et al., 2020). Based on the intercalating dye (PMA) assay, Moon et al. (2020) demonstrate that treatment with 0.2 mM of PMA (Biotium, Hayward, CA, United States) is optimal for quantifying with minimum damage to the intact HuNoV viral particle. PMA dye was added to each viral suspension sample that was concentrated by the MBS assay. The suspension was immediately mixed with 0.2 mM PMA and incubated in the dark at 5°C for 15 min to penetrate the dye into viral particles. Photo-activation between PMA dye and viral nucleic acid was induced with a high-power LED light (45-W lamp) in a photoactivation system (PhAST Blue; GenIUL, Barcelona, Spain) at a wavelength of 460 nm for 15 min at 4°C for photoactivation. Consecutively, viral RNA extraction and quantitative RT-qPCR were conducted following the protocol detailed at the ISO 15216-1:2017 (ISO15216-1:2017., 2017). Probe (Bioneer Inc., Daejeon, South Korea), and primers (Bioneer Inc.) were used as described previously (Lee et al., 2018).

### Statistical Analysis

For the analysis of RT-qPCR response data, the experimental result was denoted by log_10_ genomic copies/μL and illustrated using the Minitab 19 (Minitab LLC). Differences among mean response values were analyzed using a one-way ANOVA in Minitab 19. Each experimental trial of the designated test sets was performed in triplicate.

## Results

### Statistical Analysis and Model Buildup

The experimental data, as determined by the BBD, are presented in Table 1, along with a comparison of the predicted and observed response variables, as an inactivation effect of HuNoV using the combination of sprayed SAEW treatment and UVC-LED irradiation. According to the experimental design illustrated in Table 1, the second order polynomial equation (*Y*_*huNoV*_) was determined for the response variable with a function of three independent parameters as presented below. The second order polynomial equation of regression analysis presenting the results of the response values on the disinfection efficacies of the HuNoV in terms of the coded values of variables is as follows:

*Y*_*HuNoV*_ = 0.915–1.410 *X*_1_ + 0.01012 *X*_2_ + 0.4037 *X*_3_ + 0.0895 *X*_1_*X_1_ + 0.000002 *X*_2_*X_2_–0.03765 *X*_3_*X_3_ + 0.0.003149 *X*_1_*X_2_–0.006 *X*_1_*X_3_–0.00111 *X*_2_*X_3_,

where X_1_, X_2_, and X_3_ are the uncoded values of the SAEW amount (μL), ACC of SAEW (ppm), and UVC-LED dose (mJ/cm^2^), respectively. Table 2 presents the statistical analysis results of the experiments evaluated using ANOVA to demonstrate the second order polynomial equation. The parameters for the reduction values of HuNoV inactivation response after treatment with sprayed SAEW and UVC-LED irradiation were estimated. According to the ANOVA results (Table 2), the *F*-value < Prob was less than 0.01 with *F*-values of 69.30, indicating an acceptable model fit and that the three experimental variables revealed a prominent effect of the sprayed SAEW treatment combined with UVC-LED irradiation on the HuNoV inactivation. Particularly, the proposed model with a considerably low *P*-value of < 0.0001 and a high *F*-value of 69.30 is significant, demonstrating that there is only a 0.01% chance that such a large “model *F*-value” could be due to noise factors. In a statistical assessment, Sahoo and Gupta (2012) demonstrate that a lower *P*-value and a higher *F*-value contribute more toward the response values of the corresponding model. In our study, both *P*- and *F*-values illustrate the importance of the variable coefficients. According to our results (Table 2), the order in which the experimental variables were involved in the response was ACC of SAEW (ppm) > UV dose (mJ/cm^2^) > SAEW amount (μL). Concerning the reduction of HuNoV population by disinfection treatment using SAEW and UVC-LED, the ACC of SAEW (ppm) revealed the most influential effects on the parameters of the reduction response value of HuNoV, with the highest *F*-value of 503.58. However, UV dose (mJ/cm^2^) and SAEW amount (μL) explained 62.05 and 15.12%, respectively, indicating that the influence on the virucidal effect was lower than that of the ACC of SAEW (ppm). With regard to the strength of interaction, the amount and ACC of SAEW were strongly affected by disinfection parameters in this second order polynomial equation with significant [*F*(18.20) and *P* (0.011)] values. A successful RSM approach of optimizing the working variables of microbial decontamination under various conditions using chemical disinfectant treatment is reported in various studies previously (Andres et al., 2016; Yawei et al., 2016; Swapnila et al., 2017). Considering the relatively different responses to the chemical disinfectant treatment under various conditions, the results of bactericidal effects in previous studies cannot be translated to virucidal effects. Furthermore, the results of bactericidal studies on the UVC-LED treatment demonstrated its effect on pathogenic bacteria. However, their data cannot be used to verify the efficacy of the combined disinfection technique on HuNoV.

**TABLE 2:** Matrix design results for the experiments performed according to the Box–Behnken experimental design for inactivation of human norovirus (HuNoV).

Source	DF*^a^*	Sum of squares	Mean square	*F*-value	*P*-value prob > *F*
Model	9	3.52882	0.39209	69.30	0.000
X_1_	1	0.08555	0.08555	15.12	0.019
X_2_	1	2.84937	2.84937	503.58	0.000
X_3_	1	0.35110	0.35110	62.05	0.001
X_1_*X_1_	1	0.02961	0.02961	5.23	0.071
X_2_*X_2_	1	0.00004	0.00004	0.01	0.936
X_3_*X_3_	1	0.08374	0.08374	17.80	0.012
X_1_*X_2_	1	0.08034	0.08034	18.20	0.011
X_1_*X_3_	1	0.00058	0.00058	0.10	0.762
X_2_*X_3_	1	0.03994	0.03994	7.06	0.045
Residual	5	0.02829	0.00566		
Lack of fit	3	0.02773	0.00924	32.98	0.030
Pure error	2	0.00056	0.00028		
***R*** ^2^ **= 99.1117**					

*^a^DF, degrees of freedom.*

### Model Modification

**FIGURE 2:**
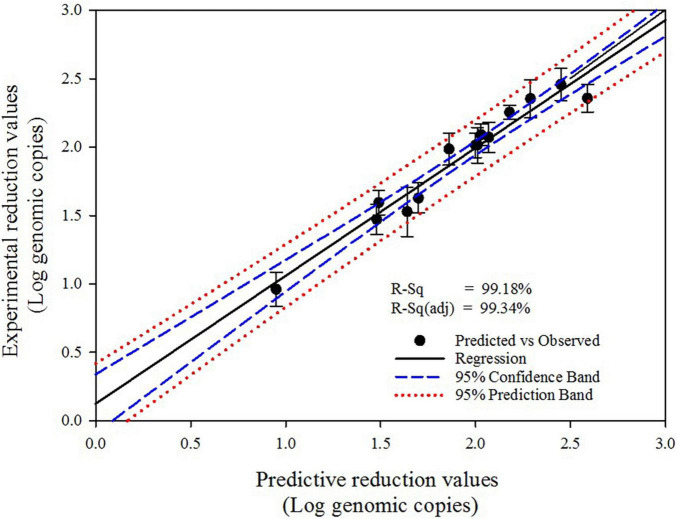
Fitted line plot presenting experimental and predicted values for the inactivation of human norovirus (HuNoV).

**FIGURE 3:**
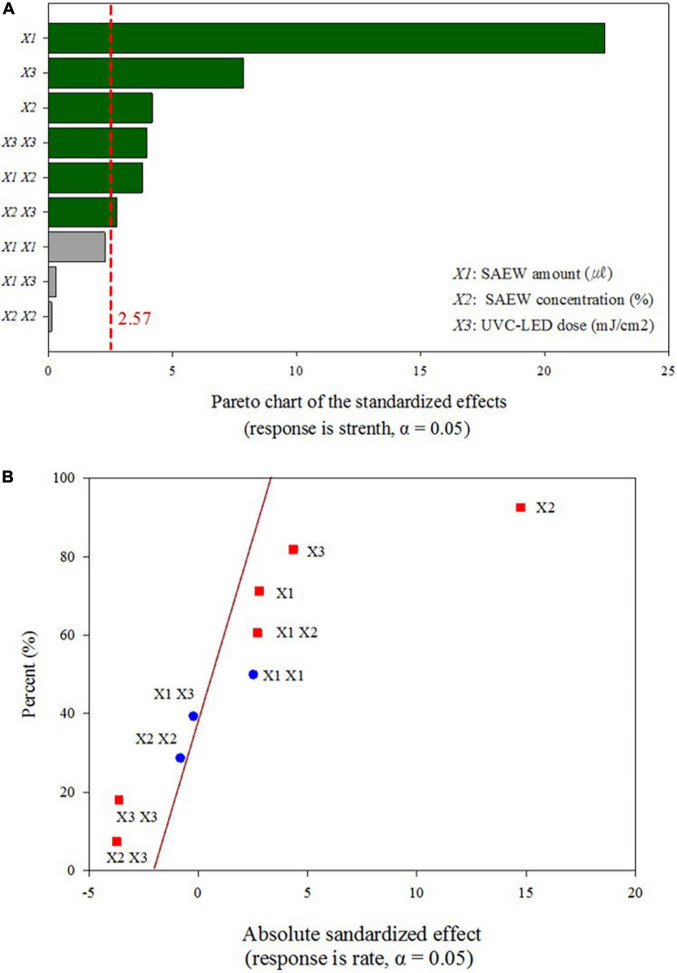
Pareto chart of standardized effects for the response values of the combination of the sprayed slightly acidic electrolyzed water (SAEW) treatment and ultraviolet C light-emitting diode (UVC-LED) irradiation at optimized disinfection conditions. **(A)** Pareto chart of standardized effects, **(B)** normal plots for standardized effects.

The significance of the proposed second-order polynomial equation was evaluated using the regression coefficient (*R*^2^) value, which presented an extremely high coefficient (0.9911) for the uniformity of HuNoV inactivation obtained under various disinfection conditions (Table 1). Moreover, the high *R*^2^ obtained for the comparison of the experimental and predicted reduction values (*R*^2^ = 0.9918; Figure 2) indicates that this second order model can be used to predict the optimized experimental conditions for the sprayed SAEW treatment and UVC-LED irradiation. The *R*^2^ (0.9918) for the sprayed SAEW and UVC-LED irradiation implies that only 0.82% variation could not be explained by the proposed second order model. The Pareto analysis identified the HuNoV inactivation variables (SAEW amount, ACC of SAEW, and UVC-LED dose), which had the greatest impact on the efficiency and reliability of a coded disinfection treatment. The relative importance of the effects of SAEW treatment and UVC-LED irradiation on HuNoV inactivation is illustrated in the Pareto chart presented in Figure 3A. According to Song et al. (2021), the Pareto analysis, demonstrating the absolute value of the standardized influence factor, is used to determine the magnitude and importance of the response effect among the independent parameter, second order, and interaction effects. The vertical red baseline Pareto chart indicates a value of 2.57 with a 95% confidence level, whereas the horizontal bar chart indicates the analyzed *t*−values. Based on a comprehensive statistical analysis, the proposed model can be used to predict the optimal disinfection conditions for HuNoV inactivation. The results of disinfection demonstrate that the ACC of SAEW (ppm) was the main virucidal parameter and the influence of the dependent variables and their interactions were ranked as ACC of SAEW (X_2_) > UVC-LED dose (X_3_) > SAEW amount*ACC of SAEW (X_1_*X_2_) > UVC-LED dose*UVC-LED dose (X_3_*X_3_) > SAEW amount (X_1_) > ACC of SAEW*UVC-LED dose (X_2_*X_3_) (Figure 3A). The normal plot of the absolute standardized effects, i.e., significant parameters and interactions as a function of the standardized effects, is plotted in Figure 3B. The normal plot of absolute standardized effects is more accurate in separating the significant and non-significant response values, and the Pareto analysis can compare the absolute values of the effects of each factor or interaction of the BBD (Secula et al., 2013). In our results, the ACC of SAEW (X_2_) and the UVC-LED dose (X_3_) reveal significant deviations from the normal distribution, and additional significant parameters include interaction between SAEW amount and ACC of SAEW (X_1_*X_2_). The brown diagonal line shows a response that occurs when a factor changes from one level to another, and “non-significant data points” indicate that the points have no influence. Effects that are further from zero are statistically significant. On this plot, red squares, including SAEW amount (X_1_), ACC of SAEW (X_2_), UVC-LED dose (X_3_), SAEW amount*ACC of SAEW (X_1_*X_2_), ACC of SAEW*UVC-LED dose (X_2_*X_3_), and UVC-LED dose*UVC-LED dose (X_3_*X_3_) parameter are retained as significant factors at the α = 0.05 level.

### Recovery Rate of HuNoV

To evaluate the elution recovery efficiency of HuNoV from the stainless-steel surface and recovery efficiency of the MBS/PMA/RT-qPCR assay, we first investigated the recovery rates from artificially inoculated surfaces and suspensions with HuNoV (approximately 5.58 log_10_), sequentially. As shown in Supplementary Figure 1, HuNoV was efficiently eluted from the stainless-steel surface when combined with the Enviro-Max Environmental sampling cotton swab and 0.05 M Glycine-0.14 M NaCl (pH 7) with mean recovery rates of 91.8 ± 1.3%. The mean recovery quantities of NoV determined using the MBS/PMA/RT-qPCR assay were 83.1 ± 2.5% for NoV (Supplementary Figure 1).

### Parameter Optimization Using Response Surface Plots

**FIGURE 4:**
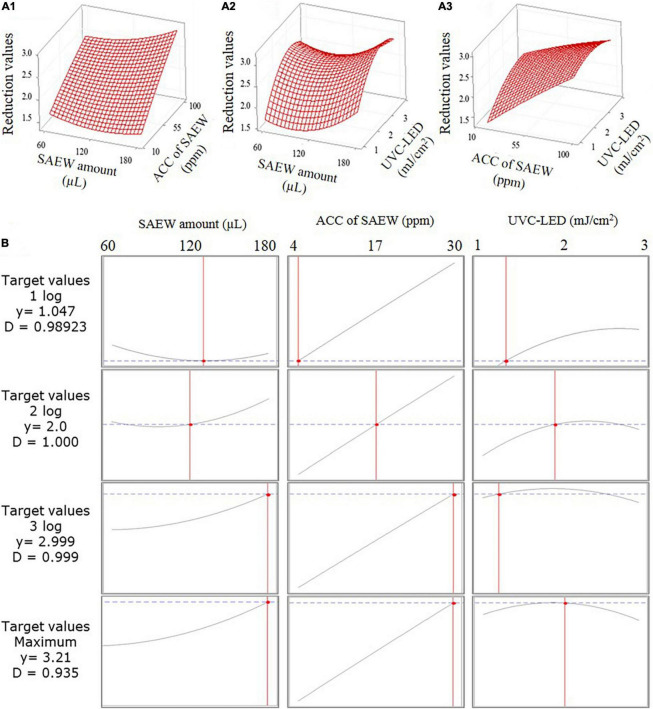
3-D response surface plots for the inactivation of human norovirus (HuNoV) using the combination of the sprayed slightly acidic electrolyzed water (SAEW) treatment and ultraviolet C light-emitting diode (UVC-LED) irradiation presenting the interaction between process parameters: **(A1)** SAEW concentration and SAEW amount, **(A2)** UVC-LED dose and SAEW amount, and **(A3)** UVC-LED dose and SAEW concentration. **(B)** Calculated optimizing plot using the MINITAB 19 software profile optimizer tool for combining the sprayed SAEW treatment and UVC-LED irradiation.

The 3-D response surface plots for parameter optimization can be illustrated as a function of two parameters while maintaining all other parameters as fixed. The obtained response surface plots are useful for understanding the interaction response values of the two types of parameters. The response values of the SAEW amount compared with the ACC of SAEW, the SAEW amount compared with the UVC-LED dose, and the ACC of SAEW compared with the UVC-LED dose using the SAEW treatment and UVC-LED irradiation for the disinfection of HuNoV are presented in Figure 4A. The optimal plot for the target response values is illustrated in Figure 4B, which presents the individual and composite desirability (*y*) and the effect of each parameter (columns) on the corresponding responses. Additionally, the individual optimal parameters for target response values (i.e., 1 log, 2 log, 3 log, and maximum log reduction value of HuNoV) are listed in Table 1. The predicted target response values were calculated by optimizing the plot using the MINITA^®^B v19 profile optimizer tool to determine the optimal parameter values for inactivating HuNoV by combining the sprayed SAEW treatment and UVC-LED irradiation. As presented in Table 3, as the target response values increased, the optimal conditions of disinfection parameters changed. When the target reduction value was increased from 1 log to 3.21 log (MAX), the change ratio in the ACC of SAEW was the largest. The proposed model predicts the maximum reduction values of 3.21 log_10_ genomic copies by the combined treatment of SAEW and UVC-LED disinfection, which were determined at 180 μL of SAEW, 30 ppm ACC of SAEW, and 2 mJ/cm^2^ UVC-LED (Table 3). The change in the ACC of SAEW changed in proportion to the target reduction value, whereas the changes in the SAEW amount and UVC-LED dose revealed a different pattern in the change of the optimal value corresponding to the target response value.

**TABLE 3:** Target response values according to combined disinfection conditions using sprayed slightly acidic electrolyzed water (SAEW) treatment and ultraviolet C-light-emitting diode (UVC-LED) irradiation.

Response values (log_10_)*^a^*	SAEW amount	ACC of SAEW	UVC-LED dose
1.00	130	20	1.3
2.00	120	55	1.9
3.00	180	100	1.2
3.21*^b^*	180	100	2.0

*^a^Target HuNoV log_10_ genomic copies reduction values.*

*^b^Maximum reduction value.*

## Discussion

HuNoV GII.4 is recognized as a major cause of acute gastroenteritis outbreaks, and sporadic illnesses caused by HuNoV GII.4 are reported (Hartmann et al., 2015). To eliminate HuNoV contamination, a potentially contaminated matrix should be disinfected properly using various methods. To this end, establishing precise qualitative and quantitative assays with improved speed and accuracy as compared with cell culture–based methods is required. In addition, there is a growing awareness of the contribution of anionic polymer as a technical tool for viral particle capture. Anionic polymer [e.g., poly (methyl vinyl ether- maleic anhydrate)] and its derivatives are copolymers with molecular and physicochemical properties that enable their application as bio-adhesives (Nunez et al., 2000). In water, such polymers dissolve as they exist in the form of ionized free acids. Polar polymer free acids are highly negatively charged in water and can be used to impart anionic charge after grafting anionic polymers to magnetic beads. They also have numerous reactive groups and can efficiently trap biomolecules, including glycine, phosphatidylcholine, oligonucleotides, enzymes, and calcium. Although the mechanism via which Poly (methyl vinyl ether-maleic anhydrate)-coated magnetic beads bind to viral particles is still unclear, components on the viral surface, such as proteins, lipids, and sugar chains, may contribute to the binding process (Sakudo et al., 2009).

There is a growing consensus that adequate and efficient disinfection, in addition to preemptive prevention of fomites contaminated with viral pathogens, are indispensable in certain environments, including hospitals, nursing homes, schools, food industries, hotels, and complex multifacilities (Koide et al., 2011). Our findings in the present study suggest that optimal disinfection conditions for HuNoV inactivation can substantially prevent exposure to the risk of viral infection. In particular, a combination of the SAEW disinfection technique demonstrated in numerous previous studies and UVC-LED irradiation technology, which has recently begun to attract attention, effectively control HuNoV GII.4, which is present on stainless-steel surfaces and is the main transmission routes. Maximum reduction effects were sufficient to eliminate the HuNoV GII.4 by combining the SAEW treatment and UVC-LED disinfection in the present study because a requirement of ≥ 3 log_10_ reduction against infectious viral pathogens, such as HuNoV, hepatitis A virus, and rotavirus, has been considered acceptable for virucidal effects (Gulati et al., 2001). Moreover, Marks et al. (2000) demonstrate that a disinfection treatment is successful in disinfecting fomites carrying pathogenic viruses if it eliminates > 3 log_10_ of the viral population, considering the titer of pathogenic viruses spread into the environment. Notably, statistical analysis of the main virucidal effects of the combination of two disinfection techniques confirms that the effect of SAEW treatment is more significant than that of UVC-LED irradiation.

Additionally, SAEW plays an important role in the inactivation of bacterial pathogens, and there is sufficient evidence of the inactivation of infectious viruses in contaminated fomites (Wigginton et al., 2012; Hakim et al., 2015). The potential mechanisms underlying the SAEW disinfection of pathogenic viruses could include (1) destruction of the virus envelope, (2) damage of the surface protein, (3) inactivation of viral nucleic acids encoding enzymes, and (4) destruction of viral RNA (Hanson et al., 1989; Morita et al., 2000; Kitano et al., 2003; Sakurai et al., 2003). The virucidal efficacy of SAEW has been investigated in various viruses, including enteric, human immunodeficiency, hepatitis B, and herpes simplex viruses (Morita et al., 2000; Tagawa et al., 2000; Park et al., 2007). In the cases of HuNoV GII.4 in suspension and on stainless-steel surfaces, the virucidal effect of 250 ppm neutral electrolyzed water treatment has been evaluated and its effectiveness verified (Moorman et al., 2017). In addition, SAEW’s capacity to inactivate HuNov has been evaluated and significant virucidal effects observed (Park et al., 2007). Meanwhile, in the context of the COVID-19 pandemic, there has been increasing attention on the requirement of immaculate disinfection techniques in food manufacturing facilities and large public places. However, there is a lack of evidence related to disinfection techniques for the virucidal efficacy of HuNoV; however, ULV sprayer disinfection is a notable approach for blocking surface-based viral particle propagation. The efficiency of several technical methods, particularly the virucidal efficacy of the SAEW sprayer against HuNoVs on various fomites, has not been investigated although ULV spraying techniques for disinfection have numerous potential advantages. In virucidal studies using commercial disinfectants, including hydrogen peroxide (7.5%), instead of SAEW, hydrogen peroxide droplets diffused by a fogging disinfection system achieved promising virucidal activity against feline calicivirus (FCV) by achieving 4 log_10_ reduction criteria for an antinoroviral disinfectant (Montazeri et al., 2017).

Several mechanisms (e.g., damage to the viral genome and/or damage to viral proteins) of virus inactivation by UVC irradiation are reported, and UVC-induced virucidal mechanisms vary among different types of viruses (Sano et al., 2010; Wigginton et al., 2010; Tanaka et al., 2018). For instance, first, UVC disinfection causes oxidative damage to the viral capsid protein, which is linked with the reduction of virus infectivity in norovirus and bacteriophage MS2 (Wigginton et al., 2010) and HuNoV surrogate FCV (Sano et al., 2010; Tanaka et al., 2018). Second, UVC irradiation destroys MNV-1 viral capsid protein. The reduction of poliovirus infectivity is reported to be secondary to viral protein-genomic crosslinking by UVC irradiation (Wetz and Habermehl, 1982). UVC-induced damage to the viral genome is observed in influenza viruses (Nakaya et al., 2020). The mechanism of UVC activation against SARS-CoV-2 is reported to be induced by viral genomic damage without apparent effects on viral morphology and proteins (Gidari et al., 2021).

Surface disinfection technology using UVC-LED irradiation has recently been highlighted (Bhardwaj S. K. et al., 2021). Particularly, Kim and Kang (2021) report that conventional mercury lamps used for UV emission are currently being replaced by UVC-LED lamps, which have higher viral decontamination efficiency and emit light sources stably and efficiently in the disinfection wavelength range of 260–275 nm. UV dose uniformity with respect to time and area is the most significant variable, which must be considered before employing UVC-LED irradiation (Araud et al., 2020). Furthermore, the fomite type should be considered an important variable because irradiation efficiency differs based on whether the fomites are porous (e.g., clothes and papers) or non-porous (e.g., glass, stainless steel, plastic) Bhardwaj J. et al. (2021). Moreover, (Bhardwaj J. et al., 2021) demonstrate that non-porous fomites are the main contributors to the transmission of viral infection because the survival duration of infectious viruses on them is much longer than that on porous fomites. To investigate the survivability of HuNoV, Ha et al. (2015) report that HuNoV GII.4 exists on artificially contaminated stainless-steel surfaces for up to 4 weeks following artificial inoculation. Previous studies have already investigated the use of UVC light to disinfect noroviruses (Jean et al., 2011; Bosshard et al., 2013). Although it cannot be compared with the direct virucidal effect of UVC-LED on HuNoV, studies of human enteric virus surrogates in a water disinfection system have been conducted to determine the virucidal effects of UVC-LED. Kim et al. (2017) report that populations of human enteric virus surrogates, including MS2, *upphi*X174, and Qβ, of the three viruses significantly decreased as the UVC-LED dose increased. Particularly, the virucidal efficacy of UVC-LED against human enteric virus surrogates was significantly inactivated by up to 7 log_10_ following treatment with 1 mJ/cm^2^ for ΦX 174 and 9 mJ/cm^2^ for Qβ and MS2 (Kim et al., 2017). For UVC-LED disinfection of SARS-CoV-2 on a rubber surface, Trivellin et al. (2021) study the design, characterization, and validation of UVC-LED disinfection and demonstrate that a 3 log_10_ reduction could be achieved following 60 s of irradiation with a 8.31 mJ/cm^2^ dose. Although UVC-LED disinfection can effectively inactivate infectious viruses, the shortcoming of UV irradiation is that there is no durative antimicrobial activity because the DNA damage of the microbial cells caused by UV irradiation can be repaired through photoreactivation or nucleotide excision repair. Although studies on the photoactivation properties of infectious viruses are limited., hurdle technology using chemical disinfection treatment, considering the shortcomings of UV disinfection, has recently been reported (Li et al., 2018; Nyangaresi et al., 2018; Wan et al., 2020).

To the best of our knowledge, this is the only study to investigate the virucidal efficacy of the combined technique of sprayed SAEW and UVC-LED irradiation against HuNoV strain GII.4 on stainless-steel surfaces. Our experimental results reveal the effective reduction values under optimal combination conditions (180 μL of SAEW, 30 ppm ACC of SAEW, and 2 mJ/cm^2^ UVC-LED dose) for HuNoV inactivation by 3.21 log_10_ genomic copies. Our experimental study included three scientific aspects: (i) Use of HuNoV to evaluate effective and promising virucidal techniques, including SAEW and UVC-LED, simultaneously; (ii) determination of the optimal parameters and correlation among experimental variables, including UV dose, SAEW concentration, and sparked SAEW amount, based on the BBD technique combined with RSM; and (iii) evaluation of the virucidal efficacy of SAEW ULV sprayer techniques on HuNoV-contaminated surfaces. However, we cannot guarantee that our results will lead to the inactivation of HuVoV under all environmental conditions owing to the lab scale. Therefore, further research is required after scaling up the SAEW ULV sprayer and UVC-LED disinfection system to a level appropriate for application in the field.

## Data Availability Statement

The original contributions presented in the study are included in the article/Supplementary Material, further inquiries can be directed to the corresponding author/s.

## Author Contributions

HS and Y-MD conducted the experiments and were responsible for assessing the experimental data, and wrote the first draft of this manuscript. SH was responsible for assessing the experimental data. J-HH was responsible for data coordination, experimental design, analysis, data interpretation, writing, revision, and finalization of the manuscript. All authors have read and approved the final manuscript.

## Conflict of Interest

The authors declare that the research was conducted in the absence of any commercial or financial relationships that could be construed as a potential conflict of interest.

## Publisher’s Note

All claims expressed in this article are solely those of the authors and do not necessarily represent those of their affiliated organizations, or those of the publisher, the editors and the reviewers. Any product that may be evaluated in this article, or claim that may be made by its manufacturer, is not guaranteed or endorsed by the publisher.
