# Supplementary material for: Evaluation of Virucidal Efficacy of Human Norovirus Using Combined Sprayed Slightly Acidic Electrolyzed Water and Ultraviolet C-Light-Emitting Diode Irradiation Treatment Based on Optimized Capture Assay for Quantitative RT-qPCR
Source: Front Microbiol. 2022 Apr 25;13:841108. doi: 10.3389/fmicb.2022.841108 (PMC9082547; doi:10.3389/fmicb.2022.841108)

**Supplementary material**

Evaluation of virucidal efficacy of human norovirus using combined sprayed SAEW and UVC-LED irradiation treatment based on optimized capture assay for quantitative RT-qPCR

Hyeyeon Song^†^, Yun-Mi Dang^†^, Sanghyun Ha, Ji-Hyoung Ha^*^

Hygienic Safety and Distribution Research Group, World Institute of Kimchi, Gwangju 61755, Korea

Fig. S1. The elution recovery efficiency of human norovir GII.4 from stainless steel surface and recovery efficiency of the MBS/PMA/RT-qPCR assay.


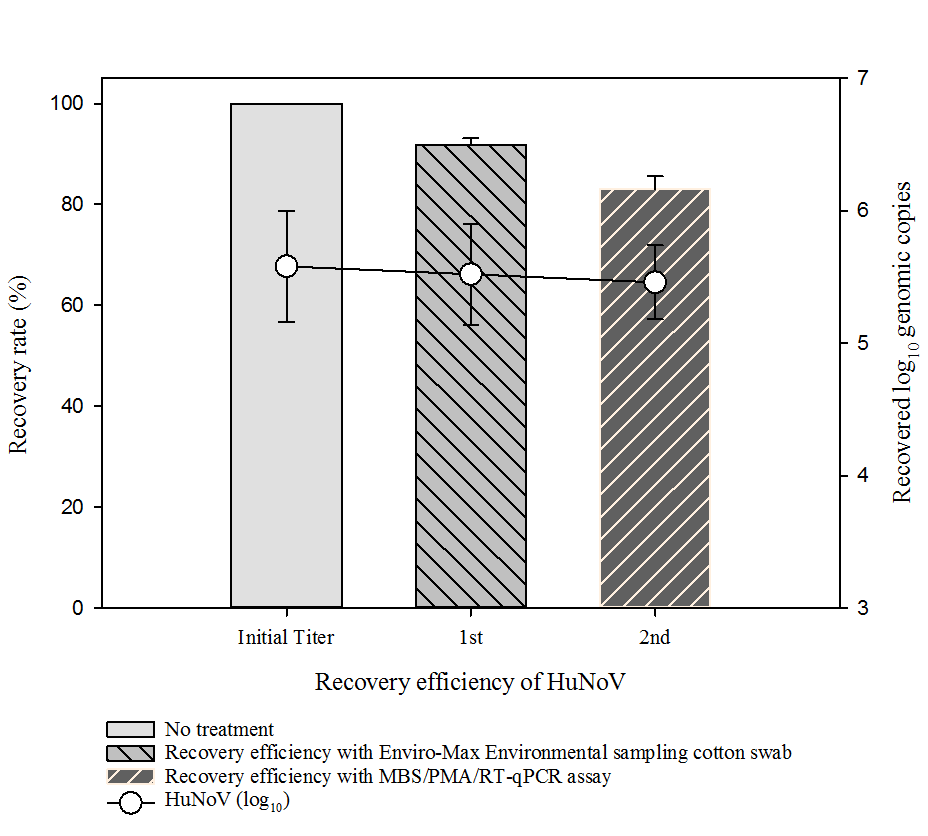

Supplement: Supplementary file 1 [file Data_Sheet_1.docx]
